# Choroidal changes after antiVEGF in neovascular age-related macular degeneration with type 1 and 3 macular neovascularization

**DOI:** 10.1007/s10792-025-03840-8

**Published:** 2025-12-05

**Authors:** Francisco de Asís Bartol-Puyal, Jorge Sánchez Monroy, Martín Puzo Bayod, Víctor Mallén, Silvia Méndez-Martínez, Pilar Calvo, Luis Pablo

**Affiliations:** 1 Miguel Servet University Hospital, Paseo Isabel La Católica, 1-3, 50009 Zaragoza, Spain; 2 Grupo Investigación Miguel Servet Oftalmología (GIMSO). Health Research Institute Aragón (IIS Aragón), Zaragoza, Spain; 3 University of Zaragoza, Zaragoza, Spain; 4 Biotech Vision SLP (Spin-Off Company), University of Zaragoza, Zaragoza, Spain

**Keywords:** Anti-vascular endothelial growth factor, Choroidal thickness, Neovascular age-related macular degeneration, Optical coherence tomography, Ranibizumab, Retinal angiomatous proliferation

## Abstract

**Purpose:**

To analyze choroidal thickness (CT) in patients with neovascular age-related macular degeneration (nAMD) with type 1 macular neovascularization (MNV) and type 3 (or retinal angiomatous proliferation) after antiVEGF treatment for two years.

**Methods:**

Retrospective study enrolling Caucasian naïve patients with nAMD (type 1 MNV), and patients with type 3 MNV with no other ophthalmological disorders, and without switching treatment. Nine manual CT measurements were performed on optical coherence tomography (OCT) Spectralis (Heidelberg Engineering). Demographic data, smoking, disease activity, and number of injections, among others were recorded. Three visits were analyzed: pretreatment baseline visit, first and last visits with no disease activity.

**Results:**

53 eyes of 53 patients with type 1 MNV, and 41 eyes of 41patients with type 3 MNV were analyzed. Both groups received the same number of injections (*p* = 0.282). Type 3 MNV patients showed lower CT (between 143.29 and 174.17μm) than type 1 MNV patients (between 169.91 and 220.17μm) in the baseline visit, but differences disappeared in first and last visits. Choroidal thinning was only observed in type 1 MNV patients between baseline and first visit (*p* < 0.05). In the las visit, they had a CT between 87 and 96% of baseline measurement. No other influencing factor was detected.

**Conclusions:**

Patients with nAMD (type 1 MNV) have higher CT than patients with type 3. However, patients with type 1 MNV experience significant choroidal thinning, and CT is similar in both groups after antiVEGF treatment. Smoking, type of drusen or other OCT features have no influence in this reduction.

## Introduction

In the last years, choroidal thickness (CT) has been deeply studied. Its distribution throughout the macula is not uniform, as seen in most published CT maps. Central and superior areas are those with higher thickness[1, 2]. Although no normality pattern has been established yet, variations in CT have been associated with different conditions and ophthalmological diseases. For example, it decreases with increasing age[3], high blood pressure[4], diabetic retinopathy[5], neovascular age-related macular degeneration (nAMD)[6], type 3 choroidal neovascularization (also known as retinal angiomatous proliferation, RAP)[7], or anti-vascular endothelial growth factor (antiVEGF) treatment[8]. It has been observed that patients with nAMD under different types of intravitreal antiVEGF treatment may experience further thinning of the choroid[9, 10]. However, it is not clear whether the combination of type 3 MNV and antiVEGF treatment implies further choroidal thinning. Previous studies in these patients found either no variation[11] or a thickening[12]. Only one study has compared subfoveal CT between patients with nAMD (MNV type 1 or 2) and type 3 MNV after intravitreal antiVEGF treatment so far[13], but CT has only been analyzed in subfoveal location yet.

CT has an importance in monitoring and predicting the evolution of patients with nAMD (MNV types 1 and 2) or type 3. On one hand, an increase in CT has been related to exudative recurrence[14, 15]. On the other hand, eyes with nAMD (MNV type 1 or 2) and retinal atrophy present lower CT than those without atrophy[16]. Thus, a better knowledge of CT variations may help us understand the underpinnings of nAMD (MNV types 1 and 2) and type 3 MNV (or RAP).

Our aim in this study is to analyze and compare CT variations in patients with nAMD (type 1 MNV) and with type 3 MNV (or RAP) after treatment with intravitreal antiVEGF for a two-year period. Secondary purpose is to find any factors which may have an influence on these variations.

## Methods

A retrospective observational study was performed at the ophthalmology department of a third-level hospital. It adhered to the tenets of the Declaration of Helsinki and was approved by the ethics committee (JZV-BEV-2018-01). Inclusion criteria were naïve patients of Caucasian race with nAMD (type 1 MNV) or type 3 MNV (or RAP) starting treatment with ranibizumab between January 2016 and December 2020, and with at least a two-year follow-up. Exclusion criteria were type 2 MNV, polypoidal choroidal vasculopathy (VCP), other causes of MNV, switching type of antiVEGF injection, presence of large subretinal hemorrhage, endophthalmitis, epiretinal membrane (ERM), glaucoma, macular hole, or best corrected visual acuity (BCVA) lower than 20 ETDRS letters (≥ 1.3 in logMAR scale). Patients were discarded in case of signs that may indicate the presence of a pachychoroid, that is, high choroidal thickness, pachyvessels with attenuation of choriocapillaris, and/or difuse leakage when performing an indocyanine green angiography (ICG). All patients were examined with Spectralis optical coherence tomography (OCT) (Heidelberg Engineering, Heidelberg, Germany). In case of bilateral affection fulfilling inclusion and exclusion criteria, only one eye was randomly selected for this study.

For differentiating type 1 MNV and type 3 MNV, fundus was assessed while indirect funduscopy, OCT was carefully evaluated, and a fluorescein angiography was performed. Type 3 MNV diagnosis was applied for those patients showing typical signs such as superficial or intraretinal hemorrhages, parafoveal intraretinal neovessels connecting to a pigmented epithelial detachment (PED), intraretinal fluid with minimal or no subretinal fluid, or retinal-choroidal anastomosis.

All patients were treated on a Treat and Extend (T&E) regime after a loading dose of three injections every month. During the two-year follow-up period, visits were recorded in the database every three months, with a margin of one month earlier or afterwards. Thus, a total of eight visits were recorded. Only three visits were taken from this database for statistical analysis. The first was the pre-treatment baseline visit (called ‘baseline visit’ from now on). The second was the first visit with no disease activity (called ‘first visit’ from now on). The third was the last recorded visit within the two-year follow-up with no disease activity (called ‘last visit’ from now on). Disease activity was assessed according to the presence of subretinal fluid, intraretinal fluid, or new retinal hemorrhage.

At the first visit, the following variables were registered: date, diagnosis (MNV type 1 or 3), gender (male or female), age, eye (right or left), smoking (non-smoker, smoker, ex-smoker), body mass index (BMI), BCVA, intraocular pressure (IOP), disease activity (absent or present), presence of retinal tubulations, presence of hard drusen, presence of soft drusen, presence of reticular drusen, presence of cuticular drusen, presence of ghost drusen, presence of drusenoid PED, presence of pseudovitelliform material, presence of PED and its height measured on OCT, presence of hyperreflective foci, presence of retinal atrophy and its extent measured on OCT, presence of subretinal fibrosis and its extent measured on OCT, presence of gray hyper-reflective subretinal exudative lesion, and presence of subretinal cleft. In consecutive visits the following data were recorded: date, number of intravitreal injections received since last recorded visit, BCVA, and disease activity.

**Fig. 1:**
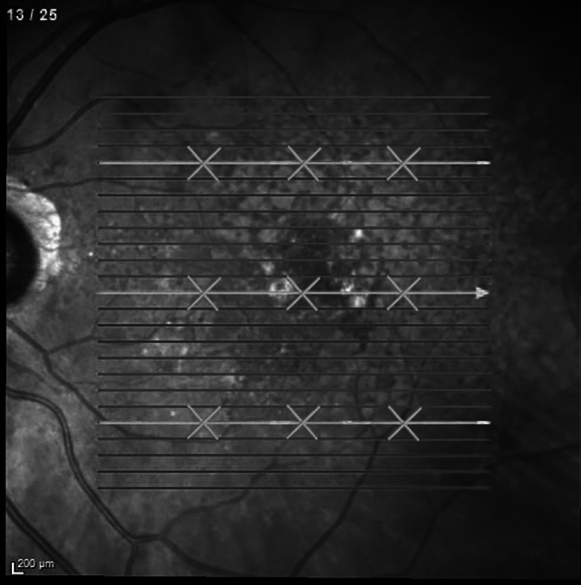
Example with the locations where choroidal thickness was measured on OCT

The same examiner manually measured CT with the integrated caliper on 9 different locations. These were central, 1500μm nasal and 1500μm temporal on OCT slabs 5/25 (inferior), 13/25 (central) and 21/25 (superior). These measurements were performed in the baseline, first and last visits. Figure 1 shows an example with the locations where CT was measured on OCT.

Statistical analysis was performed using Statistical Package for the Social Sciences (SPSS) software for Windows v.20 (IBM Corporation, Somers, NY, USA). Means and standard deviations were calculated for every quantitative variable. Chi-square test was used for comparisons between qualitative variables, Student’s t test or Student’s t test for paired data were used for comparisons between quantitative variables. Normality of variables was checked with Kolmogorov–Smirnov test. Multiple lineal regression analyses were performed afterwards. Statistical significance was established at *p* < 0.05.

## Results

**Fig. 2:**
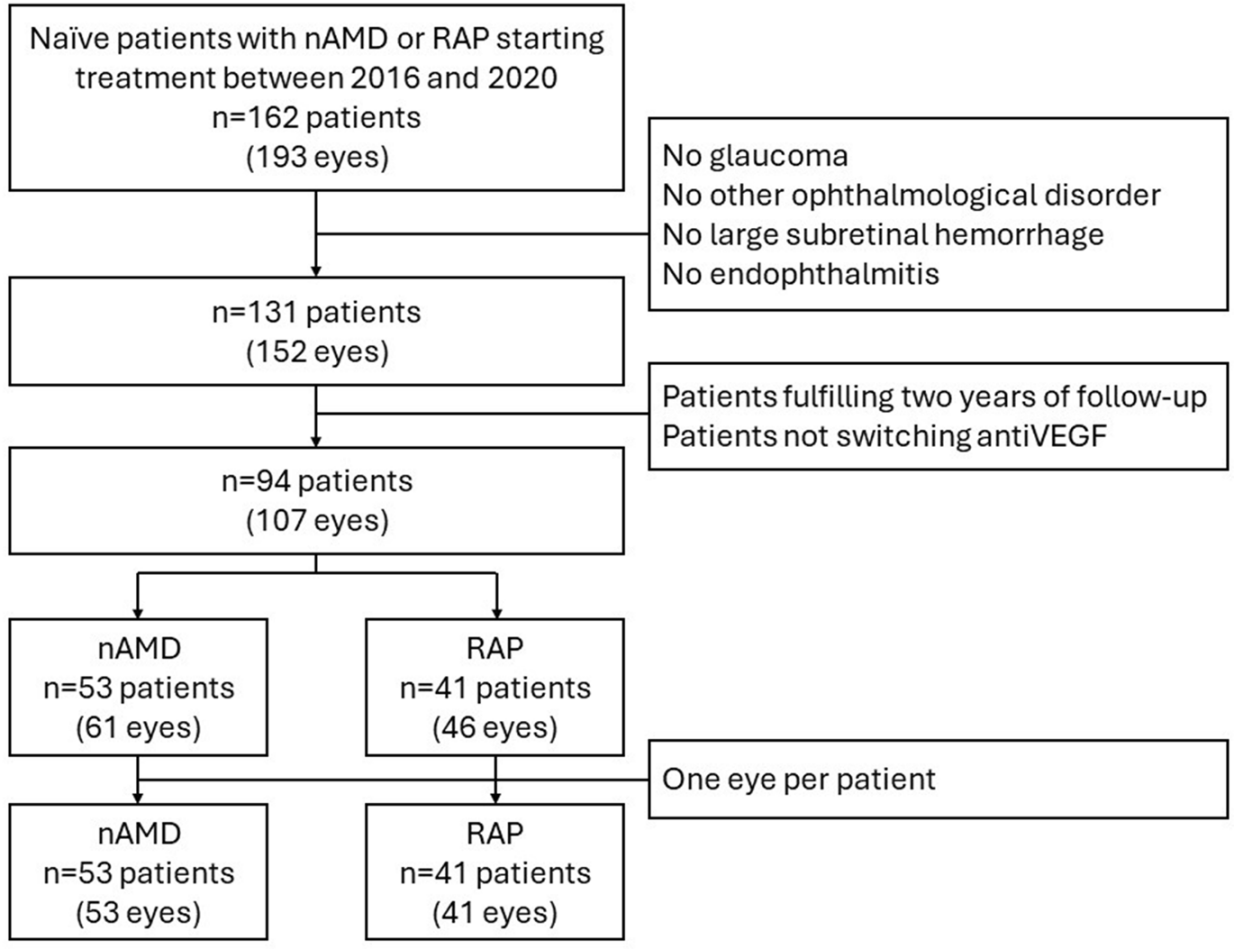
Flowchart of patients fulfilling inclusion and exclusion criteria

After applying inclusion and exclusion criteria, 53 eyes of 53 patients with type 1 MNV, and 41 eyes of 41patients with type 3 MNV were enrolled in the study. Figure 2 shows the flowchart of patients fulfilling inclusion and exclusion criteria. Within type 1 MNV group, 23 were male and 30 were female, and 27 were right and 26 were left eyes. Within type 3 MNV group, 13 were male and 28 were female, and 12 were right and 29 were left eyes. Within type 1 MNV group, there were 35 non-smokers, 10 smokers, and 8 ex-smokers. Within type 3 MNV group, there were 27 non-smokers, 1 smoker, and 13 ex-smokers. There were differences between groups due to fewer smokers in type 3 MNV group (*p* = 0.017). There were no differences in age (83.86 ± 8.22 years in type 1 MNV group, 81.48 ± 6.87 years in type 3 MNV group, *p* = 0.311), BMI (28.12 ± 5.20 kg/m^2^ in type 1 MNV group, 27.60 ± 4.64 kg/m^2^ in type 3 MNV group, *p* = 0.619), baseline BCVA (59.57 ± 15.46 ETDRS letters in type 1 MNV group, 60.83 ± 13.51 ETDRS letters in type 3 MNV group, *p* = 0.679) or IOP (15.16 ± 2.05 mmHg in type 1 MNV group, 16.23 ± 1.98 in type 3 MNV group, *p* = 0.702).

Mean time until first visit without disease activity was 8.82 ± 4.10 months in type 1 MNV group, and 7.68 ± 2.93 months in type 3 MNV group (*p* = 0.132). Before this first visit, type 1 MNV group received 4.34 ± 2.88 intravitreal injections, and type 3 MNV group received 3.80 ± 1.47 injections (*p* = 0.282).

Mean time between first and last visits was 11.31 ± 5.05 months in type 1 MNV group, and 11.28 ± 5.23 months in type 3 MNV group (*p* = 0.961). Between both visits, patients with type 1 MNV received 4.53 ± 3.15 intravitreal injections, and patients with type 3 MNV received 4.80 ± 2.36 injections (*p* = 0.640). Patients with type 1 MNV received a total of 7.68 ± 4.01 injections, and patients with type 3 MNV received a total of 7.32 ± 3.23 injections (*p* = 0.638).

Table 1 displays CT and statistical differences between both study groups in pre-treatment baseline visit, first visit with no disease activity, and las visit with no disease activity. Type 3 MNV patients showed a thinner choroid in the baseline visit in nearly all locations. However, practically all these differences disappeared in first and last visits.

**Table 1:** Mean and standard deviations of choroidal thickness (μm), and statistical differences in pre-treatment baseline visit, first visit with no disease activity, and las visit with no disease activity for a two-year follow-up

Region	Baseline visit			First visit			Last visit		
	AMD	RAP	*p*	AMD	RAP	*p*	AMD	RAP	*p*
S–N	206.19 ± 82.30	161.80 ± 60.29	**0.003**	182.68 ± 73.08	155.37 ± 67.34	**0.033**	180.83 ± 65.82	154.02 ± 65.80	0.640
S-C	206.70 ± 72.23	174.17 ± 60.15	**0.022**	197.75 ± 71.63	170.32 ± 64.44	0.067	188.42 ± 66.41	172.27 ± 67.41	**0.043**
S-T	220.17 ± 85.19	173.83 ± 62.79	**0.004**	199.53 ± 73.41	166.83 ± 61.85	**0.036**	201.38 ± 72.42	170.29 ± 65.60	0.085
C-N	169.91 ± 69.56	143.29 ± 67.21	0.065	166.32 ± 81.60	136.27 ± 72.93	0.282	150.43 ± 62.45	137.98 ± 71.29	0.638
C–C	194.77 ± 75.47	157.90 ± 59.03	**0.012**	187.60 ± 85.85	152.34 ± 70.47	0.053	173.79 ± 71.01	158.27 ± 73.39	0.513
C-T	207.55 ± 70.63	162.24 ± 55.21	**0.001**	197.36 ± 75.52	157.93 ± 67.28	0.091	186.49 ± 69.31	165.76 ± 71.24	0.774
I-N	187.72 ± 67.08	158.32 ± 76.75	0.051	177.38 ± 71.99	148.41 ± 69.77	0.132	168.53 ± 64.52	140.73 ± 65.93	0.961
I-C	197.25 ± 69.63	169.22 ± 62.94	**0.047**	188.83 ± 74.13	161.61 ± 80.00	0.139	186.87 ± 68.99	161.32 ± 72.70	0.638
I-T	195.75 ± 67.64	163.59 ± 61.16	**0.019**	191.26 ± 69.62	161.49 ± 61.24	0.472	189.13 ± 72.16	160.61 ± 73.66	0.876

S = superior, N = nasal, C = center, T = temporal, I = inferior

Statistical differences are highlighted in bold

Patients with type 1 MNV experienced a significant choroidal thinning between baseline and last visit, but not between baseline and first visit, or between first and last visit. CT in type 3 MNV patients was similar in almost all locations in the three visits. Table 2 displays these p values.

**Table 2:** Statistical differences of comparisons between choroidal thickness between baseline, first and last visits

Region	Baseline – last visit		Baseline – first visit		First – last visit	
	AMD	RAP	AMD	RAP	AMD	RAP
S–N	**0.002**	0.308	0.588	0.252	0.775	0.839
S-C	**0.008**	0.769	0.260	0.372	0.121	0.757
S-T	**0.016**	0.698	0.171	0.521	0.755	0.642
C-N	**0.022**	0.485	**0.004**	0.472	0.097	0.814
C–C	** < 0.001**	0.965	0.139	0.571	**0.008**	0.470
C-T	**0.009**	0.654	**0.007**	0.323	0.141	0.354
I-N	**0.006**	**0.004**	0.153	0.091	0.077	0.093
I-C	0.090	0.256	0.193	0.230	0.693	0.966
I-T	0.274	0.670	0.478	0.636	0.668	0.888

S = superior, N = nasal, C = center, T = temporal, I = inferior

Statistical differences are highlighted in bold

CT reductions were calculated and compared between both study groups in the three visits. Only three out of eighteen comparisons were significant. These data are displayed in Table 3.

**Table 3:** Reduction in choroidal thickness (μm) and statistical differences

Region	Baseline – first visit			First – last visit		
	AMD	RAP	*p*	AMD	RAP	*p*
S–N	25.36 ± 57.47	7.78 ± 58.22	0.957	1.85 ± 46.88	1.34 ± 42.03	0.053
S-C	18.28 ± 47.95	1.90 ± 41.23	0.197	9.34 ± 43.08	-1.95 ± 40.06	0.248
S-T	18.79 ± 54.91	3.54 ± 57.94	0.863	-1.85 ± 42.93	-3.46 ± 47.31	**0.034**
C-N	13.47 ± 41.41	5.32 ± 48.31	0.210	9.89 ± 42.52	-1.71 ± 46.11	0.185
C–C	20.98 ± 37.60	-0.37 ± 52.47	**0.034**	13.81 ± 36.67	-5.93 ± 52.08	0.303
C-T	21.06 ± 56.11	-3.51 ± 49.85	0.094	10.87 ± 52.91	-7.83 ± 53.48	0.159
I-N	19.19 ± 48.48	17.59 ± 37.28	0.865	8.85 ± 35.76	7.68 ± 28.58	**0.043**
I-C	10.38 ± 43.69	7.90 ± 43.90	0.839	1.96 ± 36.04	029 ± 43.57	0.085
I-T	6.62 ± 43.59	2.98 ± 44.36	0.873	2.13 ± 35.98	0.88 ± 39.58	0.063

S = superior, N = nasal, C = center, T = temporal, I = inferior

Statistical differences are highlighted in bold

Multiple regression analyses with dependent variable being CT reduction showed no significant outcomes in any choroidal location. In other words, none of the recorded variables showed any influence on CT variations. These independent variables were gender, age, smoking, BMI, IOP, retinal tubulations, hard drusen, soft drusen, reticular drusen, cuticular drusen, ghost drusen, drusenoid PED, pseudovitelliform material, PED and its height, hyperreflective foci, retinal atrophy and its extent, subretinal fibrosis and its extent, gray hyper-reflective subretinal exudative lesion, and subretinal cleft.

## Discussion

In this study we analyzed and compared variations in CT in patients with nAMD (type 1 MNV) and type 3 MNV (or RAP). All patients were naïve and had no other ophthalmological disorders, so variations in CT may be expected only because of the antiVEGF treatment and the disease itself. In addition, all of them were treated with the same antiVEGF, and they were discarded in case of switching. As far as we know, CT in different macular locations has not been studied yet, and only subfoveal CT has been compared between eyes with nAMD (MNV type 1 or 2) and type 3 MNV after treatment with intravitreal antiVEGF. We compared temporal, central and inferior CT in superior, central and inferior locations.

Although automatic measurements may be more accurate than manual measurements, Spectralis OCT has a very good repeatability and reproducibility in manual measurements in healthy subjects and in patients with retinal disorders[17, 18]. In case of pathologies with a pachychoroid, it would be expected that the enhanced depth imaging (EDI) mode needed activation. Nevertheless, nAMD (MNV type 1 and 2) and type 3 MNV are featured by a thinned choroid, and therefore its boundaries were clearly distinguished in all OCT slabs. We performed measurements in nine different locations because previous studies have reported differences in CT variations depending on the location[2, 5]. We only analyzed CT in those visits with no disease activity. It is very important because current knowledge indicates that reactivation of nAMD (MNV type 1 or 2) or type 3 MNV may imply an temporary increase in CT[14, 15].

CT values in eyes with type 1 MNV in our study are in line with existing literature. Most of previously reported thicknesses are between 150 and 250μm. Eyes with type 3 MNV usually present lower CT values[13]. We found that patients with type 1 MNV presented higher values of CT than patients with RAP before starting antiVEGF treatment. However, these differences disappeared when disease activity was halted for the first time, and this situation remained similar at the end of the two-year follow-up period. This fact was due to the significant choroidal thinning that patients with type 1 MNV experienced with intravitreal antiVEGF. It did not occur in patients with type 3 MNV, whose CT remained similar throughout the entire follow-up. We observed that CT after two years were between 87 and 96% of initial values in patients with type 1 MNV, and between 88 and 100% of CT in case of type 3 MNV. These percentages are similar to previously reported with the same type of antiVEGF in Caucasian and Japanese ethnicities[19, 20].

We found either no differences in the number of antiVEGF injections, nor in the time needed to stop disease activity for the first time. Between the first and the last visit with no disease activity, we found neither any differences in the number of injections received or the time that passed between both. Therefore, we believe that the different choroidal response to intravitreal antiVEGF may be because patients with type 3 MNV have already a very thinned choroid. Little variations in CT are sometimes difficult to be measured with OCT, and statistical tests usually require bigger sample sizes to show significance. Between baseline and last visits, we found a mean decrease between 0 and 17 μm in eyes with type 3 MNV, which similar to the minimal image resolution of Spectralis OCT (around 7μm)[21]. In contrast, mean CT reduction between the same visits in patients with type 1 MNV was between 6.62 and 25.36 μm.

Other authors reported similar outcomes to ours regarding type 1 MNV. Previous studies compared subfoveal CT, and found that it decreased after antiVEGF treatment for twelve months[9, 10, 22–25]. Although most studies have found a reduction, some other reported no changes[8]. The great majority of these studies focused only on subfoveal CT. Nonetheless, the choroid may respond differently depending on the location[2]. This is why it is important to analyze different macular locations.

Studies addressing CT decrease in patients with type 3 MNV are far fewer than those with MNV types 1 or 2. Kim et al. analyzed subfoveal CT in eyes with type 3 MNV and found choroidal thinning after intravitreal antiVEGF[11]. In contrast to our outcomes, Maruko et al. found that CT reduced approximately 85%, but patients included in their study had received antiVEGF and photodynamic therapy[12]. This difference may be attributed to photodynamic therapy, which causes choroidal remodeling. Tamashiro et al. compared subfoveal CT after treatment with brolucizumab, and found that it significantly decreased[20]. Nevertheless, they only performed a three-month follow-up, the kind of antiVEGF was different to that in our study, and they only measured subfoveal CT.

Little research has been performed regarding non-subfoveal CT. In general, the vast majority of previous studies report a reduction of CT, although most of them analyzed only nasal and temporal locations. Altunel et al. evaluated CT at nasal and temporal 1000 μm from fovea after three monthly intravitreal antiVEGF injections[26]. Despite differences regarding methodology, their outcomes are similar to ours. Mean CT decrease was similar between nasal and temporal measurements in central macula. Gharbiya et al. performed a similar study, but measuring CT in different locations. In contrast to our outcomes, they found greater CT reduction in patients with type 3 MNV. The main difference to our study is that their comparisons between types of MNV only considered subfoveal CT. Other locations were analyzed all together without considering the type of MNV. Koizumi et al. assessed CT reduction after a three-month treatment in patients with type 1 and type 2 MNV[27]. Their outcomes are similar to ours, with mean reductions around 20 μm in non-subfoveal locations. Mazaraki et al. also studied CT reduction in different macular locations[28]. They found that naïve patients experienced greater reductions than pretreated ones, as well as we did. They report similar reductions to ours, with no major difference between nasal and temporal. However, they did not analyzed superior or inferior macula.

Some other authors report CT reduction after antiVEGF treatment in AMD in nasal and temporal macula, as well as we found. Nonetheless, their different methods do not allow a direct comparison to our study. For example, they compare AMD with polypoidal choroidal vasculopathy, they measured CT regardless the presence of signs of activity on OCT, or they did not analyzed in detail non-subfoveal CT[22, 24].

We tried to detect possible factors influencing this choroidal reduction, but we found no significant outcomes. Although smoking was more frequent in type 1 MNV patients, it had no influence on their greater choroidal thinning. Retinal features on OCT had no influence either. These included all types of retinal drusen, retinal tubulations, PED, hyperreflective foci, or subretinal cleft, among others.

Some previous studies tried to correlate the number of antiVEGF injections and CT decrease. Similar to our outcomes, they could not find any correlations between both[29]. Kandani et al. conducted a study somehow similar to ours, in which they compared CT between nAMD (type 1 MNV), type 3 MNV and VCP. They found that CT decreased in the three pathologies, but eyes with type 3 MNV were those which decreased the most, which is just opposite to our outcomes. Differences to our study are that they only measured subfoveal CT, and their follow up was twelve months shorter than ours[13].

The strengths of our study are that all the enrolled patients were naïve, with no other ophthalmological disorder and received always the same intravitreal treatment. In addition, we compared nine macular locations different than central. The main weaknesses are our sample size and that CT measurements were manual instead of automatic.

In conclusion, patients with nAMD (type 1 MNV) have a thicker choroid than patients with type 3. However, patients with type 1 MNV experience significant choroidal thinning, and CT is similar in both groups after antiVEGF treatment. Smoking, type of drusen or other OCT features have no influence in this reduction.

## Data Availability

No datasets were generated or analysed during the current study.
